# Proteomic Changes in Induced by Fludioxonil

**DOI:** 10.1021/acs.jproteome.5c00038

**Published:** 2025-06-13

**Authors:** Pedro Henrique Corteletti Manfio, Wilson Dias Segura, Marina Valente Navarro, Rogéria Cristina Zauli, Patricia Santos Lopes, Solange M. T. Serrano, Alison Felipe Alencar Chaves, Wagner Luiz Batista

**Affiliations:** † Department of Pharmaceutical Sciences, 505146 Federal University of São Paulo, Diadema, São Paulo 09913-030, Brazil; ‡ Department of Microbiology, Immunology and Parasitology, 28105 Federal University of São Paulo, São Paulo, São Paulo 04023-062, Brazil; § Laboratory of Applied Toxinology, Center of Toxins, Immune-response and Cell Signaling, 534834 Butantan Institute, São Paulo, São Paulo 05503-900, Brazil

**Keywords:** Paracoccidioides brasiliensis, fludioxonil, proteomic analysis, cell wall proteins

## Abstract

is a pathogenic fungus capable of thermodimorphic transition, causing systemic mycoses in humans. Fludioxonil, a phenylpyrrole fungicide, inhibits this transition and affects genes related to fungal cell wall composition. This study explored the proteomic response of yeast cells exposed to fludioxonil, identifying over 4100 proteins, approximately 50% of the total proteome, through DIA (data-independent acquisition) mass spectrometry. Treatment led to significant proteomic changes, with 28 proteins upregulated and 26 downregulated. Upregulated proteins were linked to oxidative and osmotic stress responses, including the mitochondrial stress response. HSP12, the most upregulated protein, participates in osmotic stress adaptation via the HOG1 pathway, while CsbD and Memo 1 family proteins were associated with stress response and fungal invasiveness. Fludioxonil exposure increased reactive oxygen species (ROS) production and catalase activity, suggesting oxidative stress adaptation. Additionally, mitochondrial membrane hyperpolarization was observed, indicating mitochondrial dysfunction. The treatment also triggered cell wall remodeling, with reduced levels of mannosyltransferase, β-glucosidase, and Chitinase, enzymes involved in carbohydrate metabolism, while chitin synthase levels were increased. These findings reveal that fludioxonil disrupts both mitochondrial metabolism and cell wall integrity, shedding light on its antifungal mechanism in .

## Graphical abstract

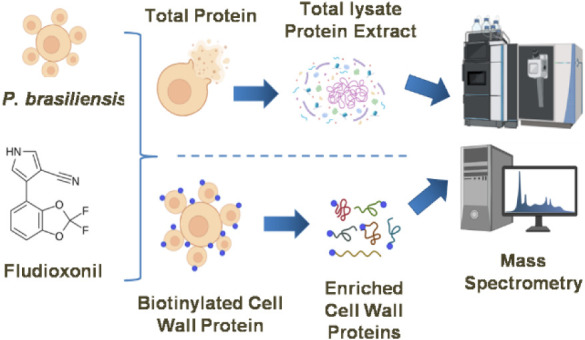

## Introduction

Paracoccidioidomycosis (PCM) is a systemic granulomatous mycosis caused by pathogenic fungi of the genus *Paracoccidioides*. PCM is geographically restricted to subtropical areas of Latin America (from Southern Mexico to Northern Argentina), with high prevalence in Brazil (80% of cases), Colombia, Venezuela, and Argentina.^1–3^ Brazil has the highest reported cases among the affected countries, with greater prevalence and mortality recorded in the southern, southeastern, and central-western regions.^4^ The disease is endemic in rural populations and primarily affects individuals involved in agricultural activities who inhale aerosols containing fungal material during soil handling.^2,5^

*Paracoccidioides* spp. is a pathogenic dimorphic and thermo-dependent fungus. At room temperature (25 °C), it presents in the mycelial form with conidia, and in parasitism or when cultured at 36–37 °C, it occurs in the yeast form. This transition capability is essential for its pathogenicity and disease establishment.^3,6^ Several fungi capable of undergoing this morphological transition are of significant medical relevance, including (), , spp., , spp., and members of the genus spp.^7^ After contagion, the initial infection usually occurs by inhalation of propagules (conidia) produced during the mycelial phase.^8^ Once inhaled, these conidia will be detected by innate immune system cells, such as macrophages and neutrophils,^4,5^ recognizing components of the fungal cell wall.

The ability to perform dimorphic transition in spp. is still poorly understood. It is known that different genes are expressed according to the fungal phase, and in recent years, genes related to the control of the mycelium-to-yeast (M-Y) transition have been identified.^9–13^ In dimorphic fungi, a dimorphism-regulating histidine kinase (Drk1) is primarily expressed in the yeast phase.^14^

Histidine kinases are proteins known as the two-component signal transduction system (TCST). This mechanism was initially described only in bacteria.^15^ The TCST system contains a conserved kinase domain (HK – histidine kinase) and a regulatory domain (RR – response regulator). In bacteria, the extracellular HK domain undergoes autophosphorylation at a histidine residue after an exogenous stimulus. This is followed by transferring a phosphate group to the regulatory domain at an aspartate residue, catalyzing a downstream reaction, and signaling a specific response.^15,16^ In fungi, histidine kinases are generally described as hybrids because they present these fused domains.^17^ A third protein, histidine phosphotransferase (HPt), is involved in the signaling pathway, promoting the transfer of phosphate from HK to RR. The signaling cascade involves four phosphorylation events: the HK domain is autophosphorylated (histidine), the phosphate is transferred to the receiver domain of the HK, a third phosphate transfer occurs to the histidine residue in the HPt domain, and finally, the phosphate group is transferred to the aspartate residue of the RR domain.^16^

Our group characterized the expression of HK (*PbDRK1*) in in a study that demonstrated its role in dimorphism promoted by molecular inhibitors of the group III hybrid histidine kinase (III HHK) iprodione or fludioxonil. In , only *PbDRK1* belongs to group III HHK. The fungus remained in the mycelial form even when cultured at 37 °C.^18,19^ Additionally, when the yeast cells of were subjected to osmotic stress, we observed high expression of *PbDRK1*, indicating a relationship with the MAP kinase Hog1 (High Osmolarity Glycerol 1) pathway in .^18^ A study by Lawry et al.^20^ suggested the existence of crosstalk between the Hog1 and Drk1 pathways in .^20^ Marcos et al.^21^ showed that down-regulating *PbDRK1* led to phenotypic alterations such as more elongated yeast cell morphology, virulence attenuation in the infection model, lower chitin content, increased resistance to caspofungin, and increased sensitivity to itraconazole.^21^

Studies involving HHK from other pathogenic fungi,^22–24^ suggest the regulation of wall components and their dimorphic capability. Navarro et al.^19^ demonstrated that fludioxonil induces the increased expression of genes involved in the synthesis and maintenance of β-(1,3)-glucan and β-(1,6)-glucan chains, such as *FKS1*, *KRE6*, *PHR2*, and *GEL3*. These results indicate that PbDrk1 regulates the expression of cell wall synthesis genes directly related to the dimorphic transition. Furthermore, there was an increase in β-glucan exposure on the surface of the fungus treated with fludioxonil.^19^ In *P. brasiliensis*, it is known that, in the fungal mycelial phase, β-(1,3)-glucans are the main components of the cell wall, while after the dimorphic transition, the main component becomes α-(1,3)-glucans.^6^ This modulation of glucan is essential for masking β-glucan molecules because the host phagocytic cells directly recognize these molecules.^25^ The ability to evade the immune response can be a critical factor in the pathogenicity and persistence of the fungus within the host.

Fludioxonil, a phenylpyrrole fungicide, is a nonsystemic analog of the antibiotic pyrrolnitrin produced by various *Pseudomonas* species.^26^ Currently, the role of fludioxonil in modulating the response to osmotic stress by the inhibition of class III HHK is well established.^26^ However, little is known about the broader cellular response and systemic effects of this compound in pathogenic fungi, especially in .

Therefore, this study aims to investigate the global proteomic response of yeast cells to fludioxonil using data-independent acquisition (DIA) mass spectrometry. By identifying differentially abundant proteins and affected cellular pathways, we aim to elucidate the mechanisms by which fludioxonil impacts fungal physiology, including oxidative stress response, mitochondrial function, and cell wall remodeling. This proteomic approach contributes to a better understanding of antifungal action at the molecular level and may help identify novel targets or biomarkers for therapeutic development. Given the limited arsenal of effective antifungals and the need for new treatment strategies against endemic mycoses, our findings are relevant in the context of antifungal drug discovery and may provide insights into the mode of action of phenylpyrrole compounds.

## Materials and Methods

### Fungal Growth and Protein Extraction

We used isolate 18 (Pb18) in our experiments. Yeast cells were cultured and maintained at 37 °C in a modified YPD (modYPD) medium (0.5% yeast extract, 0.5% casein peptone, and 1.5% glucose, pH 6.5) for 5 days.^27^ After this period, yeasts were collected, washed three times with PBS, and resuspended in 50 mL of RPMI medium supplemented with 2% glucose. The cultures were then incubated for 24 h with fludioxonil (25 μg/mL) (test condition) and without (control condition) at 37 °C with agitation at 150 rpm.

Proteins were extracted from according to the protocol of Villén and Gygi^28^ and Castilho et al.^29^ with some modifications. Cells were collected and pelleted by centrifugation (2000 × *g*, 15 min, 4 °C), rinsed three times with cold PBS, distributed into 2 mL tubes, and subjected to centrifugation at 10,000 × *g* for 5 min at 4 °C. The supernatant was subsequently removed, and cytoplasmatic proteins were prepared by homogenizing the yeast cells with glass beads (beads 425–600 μm, Sigma, St. Louis, MO, USA) in 700 μL of cold lysis buffer [50 mM Tris-HCl (pH 7.5), 2 mM EDTA, 2 mM DTT, 50 mM KCl, 0.2% Triton X-100, 1 mM PMSF, 1 tablet of protease inhibitor (Complete tablets, Roche) and 1% phosphatase inhibitor solution (Sigma, Saint Louis, USA)]. Then, the yeast cells were vortexed for lysis with five cycles of 90 s^28,29^ and centrifuged at 1000 × *g* for 3 min at 4 °C to separate the glass beads from the lysate. The supernatant was collected and centrifuged at 15,000 × *g* for 10 min at 4 °C. This experiment was conducted in quintuplicate.

### Biotinylation of Cell Wall Proteins

#### Biotin Labeling

 yeast cells were pelleted (approximately 1 mL of yeast pellet), washed, and resuspended in 3 mL of PBS containing Sulfo-NHS-LC-Biotin (Thermo Scientific) at a concentration of 1 mg/mL, and incubated for 1 h at 4 °C. After incubation, 6 mL of 100 mM Tris-HCl (pH 7.4) was added, and the mixture was incubated for an additional 30 min at 4 °C. Following incubation, the solution was centrifuged at 3000 × *g* for 5 min at 4 °C, the supernatant was discarded, and the pellet was resuspended in cold PBS, followed by centrifugation at 4000 × *g* for 5 min at 4 °C. This washing process was repeated three times.

#### Fungal Lysis for Biotinylated Protein Extraction

Biotinylated proteins were recovered following the protocol by Jia et al.,^30^ with modifications. Initially, the biotinylated yeast cells were resuspended in 2 mL of PBS containing protease inhibitors (Complete tablets, Roche) and lysed using glass beads with vigorous vortexing (10 cycles, 1 min of vortexing followed by 1 min on ice). After mechanical lysis, the supernatant was separated from the pellet by centrifugation at 16,000 × *g* for 10 min at 4 °C. The pellet was washed twice with a 1 M NaCl solution and centrifuged again at 16,000 × *g* for 5 min at 4 °C. The pellet was then washed three times with 50 mM Tris-HCl (pH 7.4), resuspended in 2 mL of extraction buffer (2% SDS, 50 mM Tris-HCl, pH 7.4, 100 mM EDTA, 150 mM NaCl, 40 mM DTT),^30^ boiled for 10 min at 100 °C, and centrifuged at 16,000 × *g* for 10 min at 4 °C. The supernatant was collected and designated as the ″SDS extract.″ The pellet was washed three times with PBS, centrifuged at 16,000 × *g* for 5 min at 4 °C, washed again with distilled water (three times), centrifuged at 16,000 × *g* for 5 min at 4 °C, and resuspended in 2 mL of 30 mM NaOH solution followed by incubation overnight at 4 °C. Then samples were centrifuged at 16,000 × *g* for 5 min at 4 °C, and the supernatant was collected and designated as the ″NaOH extract″.

#### Purification and Precipitation of Biotinylated Proteins

To purify the biotinylated proteins from , a 250 μL aliquot of the resin (Pierce Streptavidin Agarose, Thermo Scientific) was collected, centrifuged at 500 rpm for 1 min at room temperature, and the recovered resin was washed three times with 250 μL of PBS. The SDS and NaOH extracts were incubated with the resin for 1 h at room temperature with agitation at 100 rpm. After this period, the pellets were washed five times with 1 mL of PBS and centrifuged at 500 rpm for 1 min at room temperature. Finally, the biotinylated proteins were eluted with 2 mL of 8 M Guanidine-HCl solution (pH 1.5). A 200 μL aliquot of the sample was then added to 1.8 mL of 100% ethanol at 4 °C and incubated at −20 °C for 10 min. Then the samples were centrifuged at 16,000 × *g* for 5 min at 4 °C, the pellet was resuspended in 1.8 mL of 90% ethanol and centrifuged again at 16,000 × *g* for 5 min at 4 °C, and the pellet was recovered.

#### Trypsin Digestion of Yeast Proteins

, yeast cells were harvested and lysed using lysis buffer [50 mM Tris-HCl (pH 7.5), 2 mM EDTA, 2 mM DTT, 50 mM KCl, 0.2% Triton X-100, 1 mM PMSF, 1 tablet of protease inhibitor (Complete tablets, Roche) and 1% phosphatase inhibitor solution (Sigma, Saint Louis, USA)] and protein content was estimated using BCA protein assay kit (PierceTM) following the manufacturer recommendations. A total of 50 μg of proteins were reduced by 5 mM Tris­(2-carboxyethyl) phosphine (TCEP) and alkylated with 20 mM 2-chloroacetamide (CAA) for 30 min at room temperature in the dark. Samples were cleaned and digested using the single-pot, solid-phase-enhanced sample-preparation (SP3) protocol.^31^ The proportion of trypsin to substrate was 1:100 and the mixture was incubated for 16 h at 37 °C under 1300 rpm.

#### LC-MS/MS Analysis

The analysis was performed in a Vanquish Neo chromatographic system (Thermo Fisher Scientific) with a trap column and a 15 cm C18 separation column (Acclaim PepMap C18, Thermo) with a gradient of 90 min starting from 5% to 30% buffer B (80% acetonitrile, 0.1% formic acid) in buffer A (0.1% formic acid in water) at 300 nL/min for 70 min, 30 to 40% buffer B at 300 nL/min for 7 min, 40 to 99% buffer B at 300 nL/min for 12 min. The LC system was coupled to an Orbitrap Exploris 480 mass spectrometer equipped with a FAIMS source, operating in positive mode with 2.1 kV, with the capillary tube heated at 270 °C, and default charge state set to 2. The FAIMS source was operated using 2 compensation voltages at −45 and −60 V. Master scan ranged from 385 to 1015 *m*/*z*, with RF Lens at 50%, Normalized AGC target of 300%, and profile data type. The DIA analysis was set to scan range from 300 to 950 *m*/*z*, isolation window of 10 *m*/*z*, normalized stepped collision energy (HCD) of 28 and 32%, resolution of 30,000 fwhm, RF lens at 50%, normalized AGC target of 1000%, maximum injection time of 50 ms, and centroid data type.

#### Data Analysis

Raw files were converted to mzML with MSConvert software using the PeakPicking filter and loaded into the DIA-NN 1.9 search engine. The trypsin specific search was done with a reference proteome fasta file (UP000001628 with 8,399 sequences available at UniProtKB) to generate a predicted spectral library and the quantification strategy was set to high precision. Statistics were performed using R language with a quarto markdown document written specifically for the project. Differences in protein abundance were probed using limma R package with robust regression method after removing outlier samples, reducing sparsity, and imputing remaining missing values. Proteins were considered differentially abundant when adjusted *p*-value (Benjamini-Hochberg correction) was ≤ 0.05 and absolute log2 of fold change was ≥ 0.58. This decision considers significant differences with a magnitude greater than 1.5 times. Missing values were evaluated using naniar R package. Imputation was performed using recursive partitioning and regression trees implemented in dlookr R package. Multivariate analysis (PCA and PLS-DA) was performed using mixOmics R package. Overrepresentation analysis was performed using Gene Ontology terms recovered from UniProtKB and testing hypergeometric distribution for sampling without replacement with phyper function from stats R base.

#### Mitochondrial Membrane Potential Assay

Rhodamine 123 staining (Sigma-Aldrich), as previously described with minor adjustments, was carried out.^32^ cells (1 × 10^7^ cells/mL) were exposed to various concentrations of fludioxonil for 24 and 48 h at 37 °C. After treatment, the cells underwent two washes with PBS and were then resuspended in a solution containing rhodamine 123 (10 μL/mL) for 15 min at 37 °C. Subsequently, the stained cells were washed twice and resuspended in 500 μL of assay buffer for analysis by flow cytometry in FACScalibur (Becton Dickinson, New Jersey, USA), using FlowJo software, version 9.7.6 (Tree Star). A total of 10,000 events were analyzed per sample.

#### ROS Production

 yeasts (1 × 10^6^) were grown in YPDm medium and treated or not (control) with fludioxonil (25 μg/mL) for 24 and 48 h. The yeasts were incubated with 60 μM DCFH-DA (2′, 7′-dichlorofluorescein diacetate) in the dark for 30 min with gentle shaking. Then, the yeasts were collected and washed three times with PBS and applied to 96-well plates, and to ensure the efficiency of the probe, untreated yeasts were incubated for 30 min in the dark with H_2_O_2_ at a concentration of 2 mM. After this period, the fluorescence was measured using a Microplate Reader (Biotek) (excitation wavelength at 485 nm and emission at 528 nm).

#### Catalase Assay

Total catalase activity was assayed using a spectrophotometer to measure the decrease in absorbance at 240 nm^33^ immediately after preparing the yeast extracts. One unit of catalase decomposed 1 mmol of H_2_O_2_ in 1 min at 25 °C. Experiments were performed in biological triplicate, and catalase-specific activities were calculated using the extinction coefficient of 0.0394 mM^–1^ cm^–1^.^34^

## Results

### Experimental Quality Control Assessment

In recent years, our group has demonstrated the influence of fludioxonil in inhibiting the thermodimorphism of and in modulating various genes related to the fungal cell wall composition.^18,19^ In this study, we investigated the effect of fludioxonil on the proteomic profile of yeast cells. For this purpose, we cultured the yeast cells in a YPDm medium treated with fludioxonil and an untreated control medium for 24 h. The fungal cells were then collected by centrifugation and subjected to cell lysis using a protein extraction buffer. We also investigated the enriched fraction of surface proteins by biotinylation labeling. The resulting protein extracts were quantified (Figure 1). We used five replicates for each experimental condition to carry out proteomic analysis.

**Figure 1:**
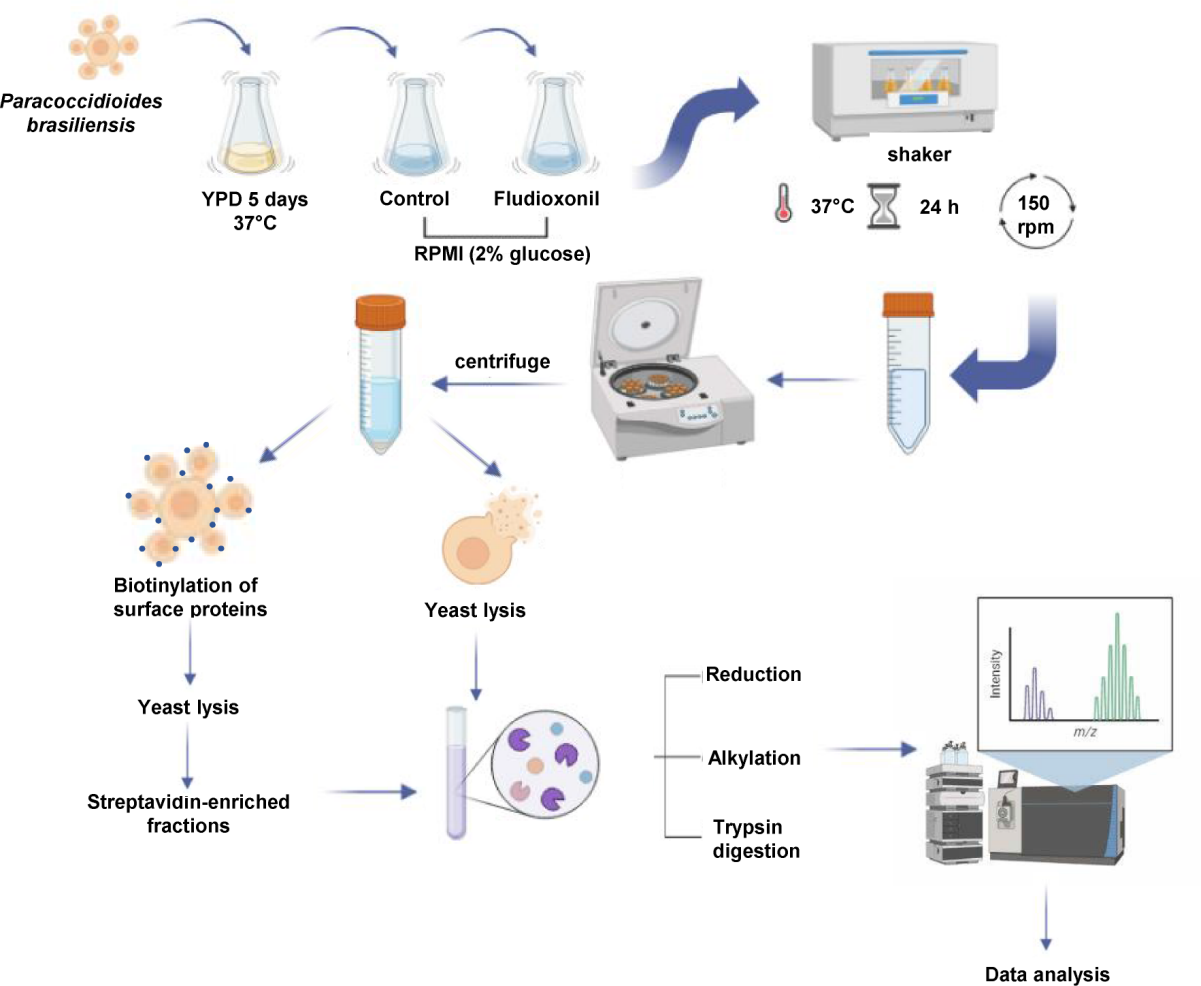
Schematic presentation of the experimental design for obtaining the proteome after exposure to fludioxonil. yeasts were grown in YPDm for 5 days at 37 °C. The yeasts were incubated in an RPMI medium supplemented with 2% glucose. The samples were treated with fludioxonil for 24 h at 37 °C. The proteins were reduced, alkylated and digested with trypsin from the total protein extract or the surface protein extract (biotinylated proteins). The peptides were evaluated in a mass spectrometer to obtain the *m*/*z* spectra. The raw files were converted to mzML with the MSConvert software using the PeakPicking filter.

While LC-MS/MS experiments can be highly informative, the data can be misleading if the quality controls are not properly assessed before statistical inferences are made. Here we show that the analysis of DIA proteomics of yeast cells treated or not with fludioxonil is reproducible and robust for quantitative study (Figure 2). The chromatographic parameter used to assess the reproducibility of sample runs is the peak signal distribution over retention time, which was as expected considering the samples are biological replicates (Figure 2A). Interestingly, the Pb18 yeast cells exposed to fludioxonil for 24 h show a remarkable signal in sample F2 at 30 min of the gradient. We checked the precursor identification responsible for this signal and found that the peptide sequence identified as the most abundant in samples F1 and F2 is ACLYAGIK.

**Figure 2:**
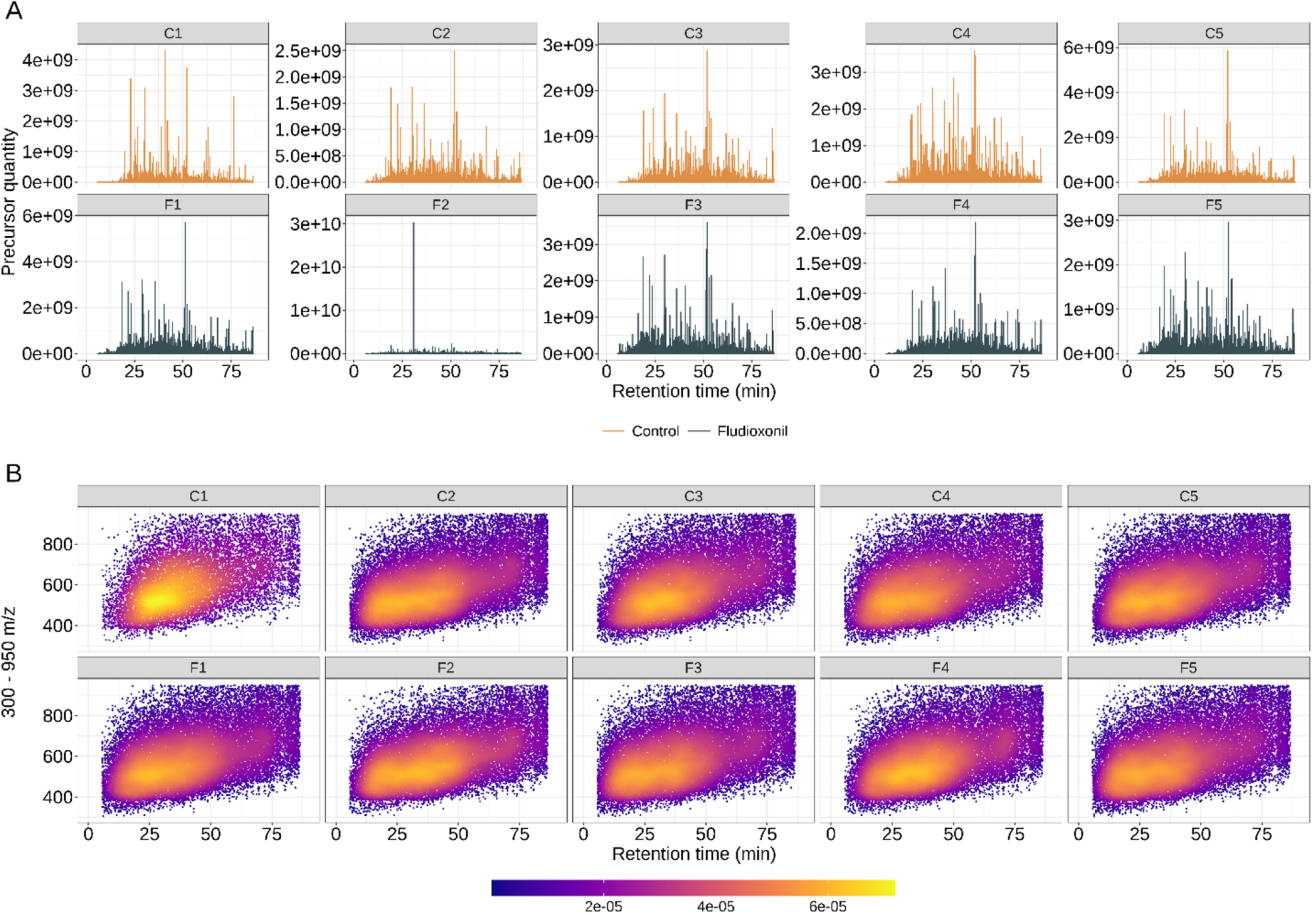
Chromatographic and mass spectrometry quality assessment. (A) Signal related to precursor quantity across the runs. (B) Scan range from 300 to 950 *m*/*z* showing the ion density across the runs along the 90 min gradient. The color bar shows the lowest (violet) density to highest (yellow) ion density over the gradient. The LC-MS/MS experiment was performed with five biological replicates.

This peptide sequence is unique for the protein glutamine synthetase (PADG_01536) in the Pb18 proteome database, which indicates the presence in high abundance of this protein. Considering that glutamine synthetase is responsible for supplying the cellular demands of glucosamine, an important component of the cell wall, it is reasonable that this protein is highly abundant after a treatment with fludioxonil. Indeed, Tomazett et al.^35^ reported an increase in transcript levels of glucosamine-6-phosphate synthase 1 h after Pb18 yeast cells exposure to SDS or KCl followed by an increase in N-acetylglucosamine content. This is a well-known response to osmotic stressors. We are aware that the glutamine synthetase is not consistently increased across the samples, but it is interesting that this enzyme is identified among the top 100 most intense ions in the fludioxonil treated cells but not from the control cells.

Further assessing the quality control of our samples, the ion *m*/*z* distribution over retention time allowed to observe that, during the sample C1 analysis, a lower number of scans was collected compared to other biological replicates. All the other samples were consistently distributed over retention time. Overall, the ion population was most distributed within the scan range 400–800 *m*/*z* and eluted during the first 60 min of the gradient (Figure 2B).

We were able to identify 4108 unique proteins in a single experiment with yeast cells, with more than 3000 proteins per sample (Figure 3A). Since the reference proteome database contains 8399 sequences, our results covered 48.9% of the canonical proteome, which represents an improvement in the field of medical mycology proteomics and opens a new avenue for future studies using a similar strategy. While the identification numbers are quite high, there are some small variations that need to be considered when performing statistical tests. In the present pipeline for data analysis, we first performed a sparsity analysis to check missingness patterns in the samples, which revealed a total of 10.3% missing values in the matrix of abundance (Figure 3B). The sparsity profile showed that sample C1 had the highest individual missingness proportion (24%) (Figure 3B), which is justified by the lower number of precursors collected (Figure 2B) resulting in less proteins identified in this sample (<3500).

**Figure 3:**
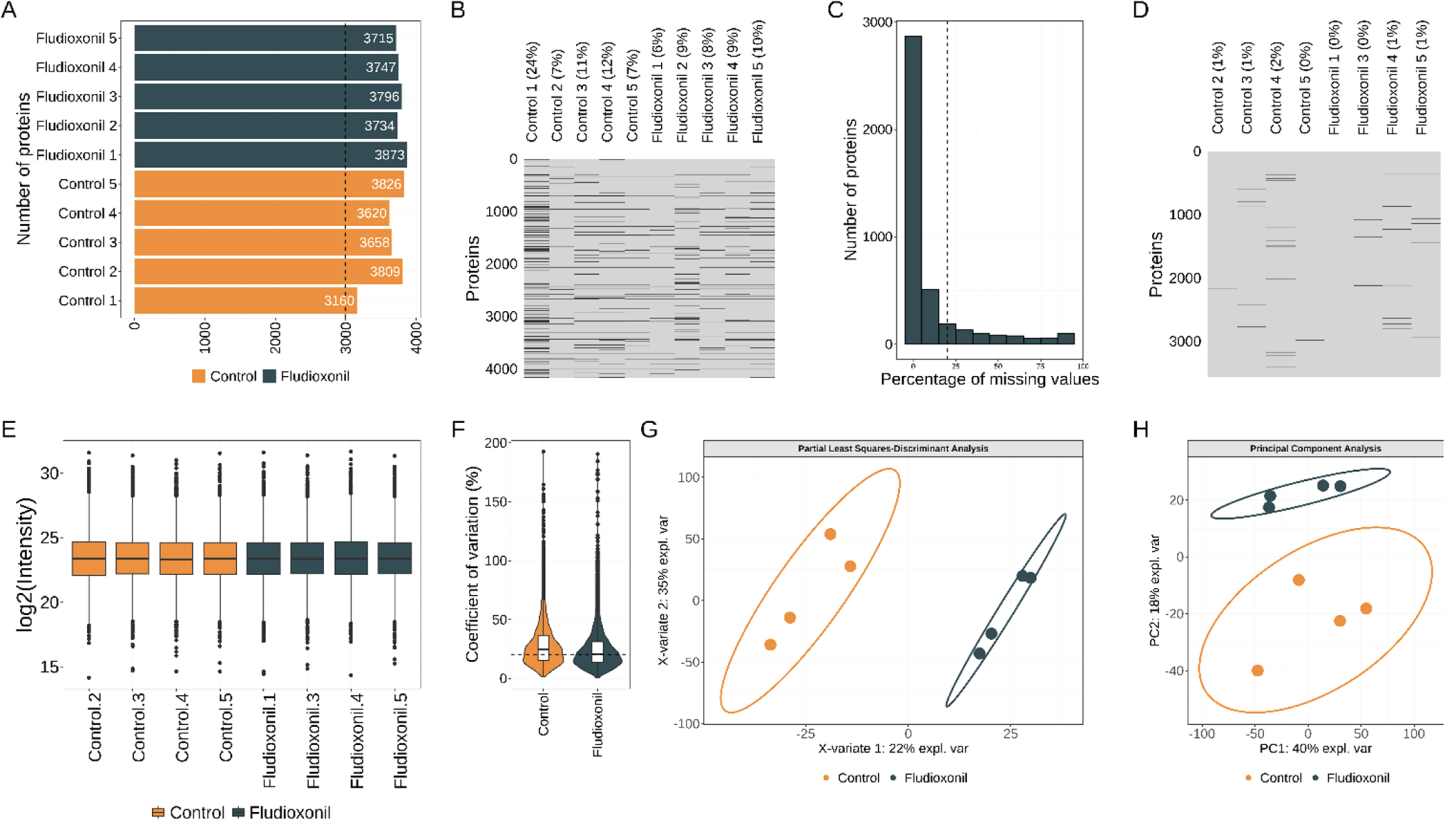
(A) Number of proteins identified per sample. Dashed line marks 3000 proteins. (B) Profile of missing proteins in the samples. Samples are shown in columns and protein numbers in rows. Gray color means presence and black color means missingness. (C) Protein distribution considering the sparsity profile. Missing values in the total data set is 10.3%. Dashed line shows the 20% cutoff for sparsity reduction per protein. (D) Missing values pattern after sparsity reduction to keep only proteins present in 20% of samples. Missing values in the total data set is 0.8%. (E) Normalized protein abundance distribution. (F) Violin plots showing distribution of the coefficients of variation across the groups. Dashed line marks 20%. (G) Multivariate analysis to classify samples in different groups using partial least-squares-discriminant analysis or (H) Principal component analysis.

To make the protein quantification robust, we balanced the groups removing the samples C1 (because of the lower number of proteins) and F2 (because of the heterogeneity in precursor abundance) and reduced the sparsity per protein keeping only those with maximum 20% of missing values (Figure 3C). After the sparsity reduction step, the matrix of protein abundance was composed of 3,567 proteins with 0.8% missing values remaining (Figure 3D). Once we had <1% missing values, we decided to impute the matrix of abundance. One important assumption that needs to be matched before imputation is that the missingness pattern is distributed at random. The Little’s missing completely at random test (MCAR)^36^ was performed under the null hypothesis that the data is MCAR, resulting in a not significant *p*-value and suggesting that the data could be properly imputed. We proceeded with log2 transformation and imputation using recursive partitioning and the regression tree (rpart) method prior to the relative quantification analysis.

After the removal of outlier samples, sparsity reduction to maximum 20% per protein, and imputation, we normalized the protein abundances to have the same median absolute deviation (Figure 3E). The median coefficients of variation for protein abundances are about 20% per group (Figure 3F), which is acceptable for a good precision in quantitative experiments. The multivariate analysis classified the samples into two groups sufficiently distinct using the supervised (PLS-DA) or unsupervised approach (PCA) based on the protein abundance profiles (Figure 3F,G). Using PLS-DA, we were able to explain 57% of total variance in the first two components, while, using PCA, we captured 58% of total variance in the first two components.

### Quantitative Proteomic Analysis of Pb18 Yeast Cells under Fludioxonil Exposure Suggests the Activation of a Response to Oxidative/Osmotic Stress

After data normalization, we fitted a linear model to the protein abundance matrix to estimate the significant differences in protein quantities between the control and fludioxonil treated Pb18 yeast cells (Table 1). Using an adjusted *p*-value cutoff of 0.05 and an effect size of absolute log2 (FC) ≥ 0.58, we found 28 proteins significantly increased (Table 1 and Figure 4A) and 26 significantly decreased 24 h after fludioxonil exposure (Figure 4A). Evaluating the enriched biological processes from decreased proteins can help to understand which pathways have been affected after fludioxonil exposure. We found that calcium-mediated signaling, biosynthesis of proline, and catabolism of threonine and serine were negatively regulated in Pb18 yeast cells (Figure 4B). On the other hand, biological processes related to mitochondria response to stress, such as protein insertion into inner membrane, cardiolipin biosynthesis, mitochondria organization, mitochondrial respiratory chain complex I assembly, protein quality control for misfolded proteins, and response to oxidative stress, such as transsulfuration were enriched in differentially increased proteins (Figure 4C).

**Table 1:** List of Major Proteins Regulated after Fludioxonil Treatment

**Gene**	**Protein name**	**Log2FC**
PADG_04907	Chaperone/heat shock protein Hsp12	4.857
PADG_07647	CsbD-like domain-containing protein	1.885
PADG_02887	Pru domain-containing protein	1.753
PADG_08406	O-acetylhomoserine (Thiol)-lyase	1.700
PADG_08334	Short chain dehydrogenase/reductase	1.626
PADG_05835	Leptomycin B resistance protein pmd1	1.536
PADG_04240	Uncharacterized protein	1.523
PADG_03031	CobW/HypB nucleotide-binding protein	1.485
PADG_08131	Uncharacterized protein	1.291
PADG_01931	Up-regulated during septation protein 1	1.268
PADG_06793	Endoplasmic reticulum transmembrane protein	1.184
PADG_00538	Meiotic sister chromatid recombination protein 1	1.114
PADG_03319	Protein translocase SEC61 complex gamma subunit	1.032
PADG_08516	AmmeMemoRadiSam system protein B	1.027
PADG_05929	Protein-L-isoaspartate O-methyltransferase	0.975
PADG_00388	Cytochrome oxidase c assembly domain-containing protein	0.947
PADG_06450	Mitochondrial large ribosomal subunit	0.940
PADG_08719	FAD-binding FR-type domain-containing protein	0.930
PADG_08554	Small ribosomal subunit protein mS29	0.929
PADG_08340	KOW domain-containing protein	0.901
PADG_04367	Amidase domain-containing protein	0.889
PADG_04687	Glucose 1-dehydrogenase	0.856
PADG_06345	AMP-dependent synthetase/ligase protein	0.835
PADG_00189	Lysophospholipase NTE1 (Intracellular phospholipase B)	0.811
PADG_06162	FYVE-type domain-containing protein	0.802
PADG_03532	Pre-rRNA-processing protein TSR2	0.795
PADG_06672	P-type Na(+) transporter	0.794
PADG_01370	Enoyl-CoA hydratase	0.768
PADG_12329	Prefoldin subunit 2	0.761
PADG_00572	Peroxisomal membrane protein PEX14 (Peroxin-14)	0.755
PADG_02227	C2H2-type domain-containing protein	0.746

**Figure 4:**
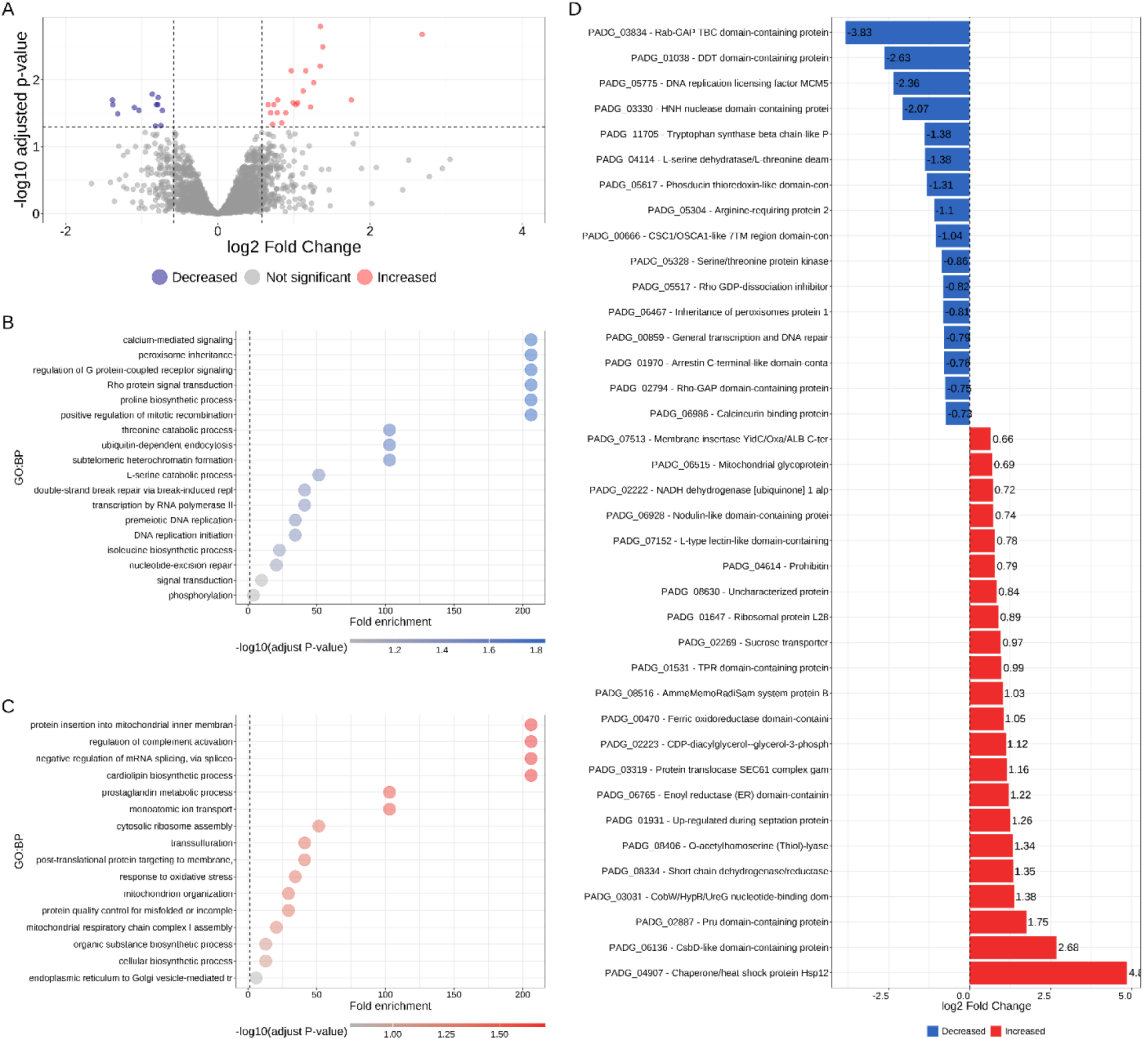
(A) Volcano plot showing the numbers of proteins with abundance significantly increased (red), decreased (blue), or not significant (gray). Dashed lines mark the cutoffs for log2­(FC) and −log10 (adjusted p-value). Overrepresentation analysis using the Gene Ontology terms recovered from (B) significantly decreased proteins (blue) or (C) significantly increased proteins (red). (D) highlight significantly differentially abundant proteins when comparing Pb18 yeast cells exposed to fludioxonil against the control yeast cells.

We show all differentially abundant proteins in Figure 4D and Table 1. Under fludioxonil exposure, the most increased protein was HSP12 (PADG_04907). This protein is overall low but is reported to have high levels of transcript upon entry on stationary phase, as well as in response to osmotic stress coordinated by HOG1 pathway.^37^ Consistent with the increase in HSP12 protein level, CsbD protein accompanied the increase after fludioxonil exposure. Under osmotic stress, CsbD is upregulated and triggers the transcription of ABC transporter genes in group B .^38^ Memo 1 family protein (PADG_08516), which has been described to be upregulated in response to stress and invasiveness phenotype in yeast,^39^ has also been found in higher abundance in Pb18 cells upon the effect of fludioxonil.

Furthermore, the protein Pleckstrin-like receptor for ubiquitin (PADG_02887) was among the most increased proteins upon fludioxonil exposure. This suggests that proteolysis was activated in the yeast cells exposed to fludioxonil and the proteasomal ubiquitin receptors were prone to detect ubiquitin labeled proteins to unfold and degrade them.^40^ The increase of prohibitin abundance in Pb18 yeast cells exposed to fludioxonil is consistent with a response to mitochondrial stress. Prohibitin abundance is reported to increase upon metabolic stress and is known by its chaperone activity over mitochondrial proteins.^41^

Overall, our results suggest that the exposure to fludioxonil triggers the oxidative/osmotic stress response that probably originated from the mitochondrial metabolism impairment. This response could cause the unfolding of proteins and degradation via proteolysis.

In addition, various proteins involved in mitochondrial metabolism were identified as upregulated. To determine whether fludioxonil caused any changes in mitochondrial membrane potential, a qualitative assay using rhodamine 123 staining was conducted. The results clearly show that after treatment with fludioxonil, there was a mitochondrial membrane hyperpolarization of approximately 3–4 times at 24 and 48 h (Figure 5A). This suggests that, at the tested time points, fludioxonil treatment induced hyperpolarization of the mitochondrial membrane in yeast cells.

**Figure 5:**
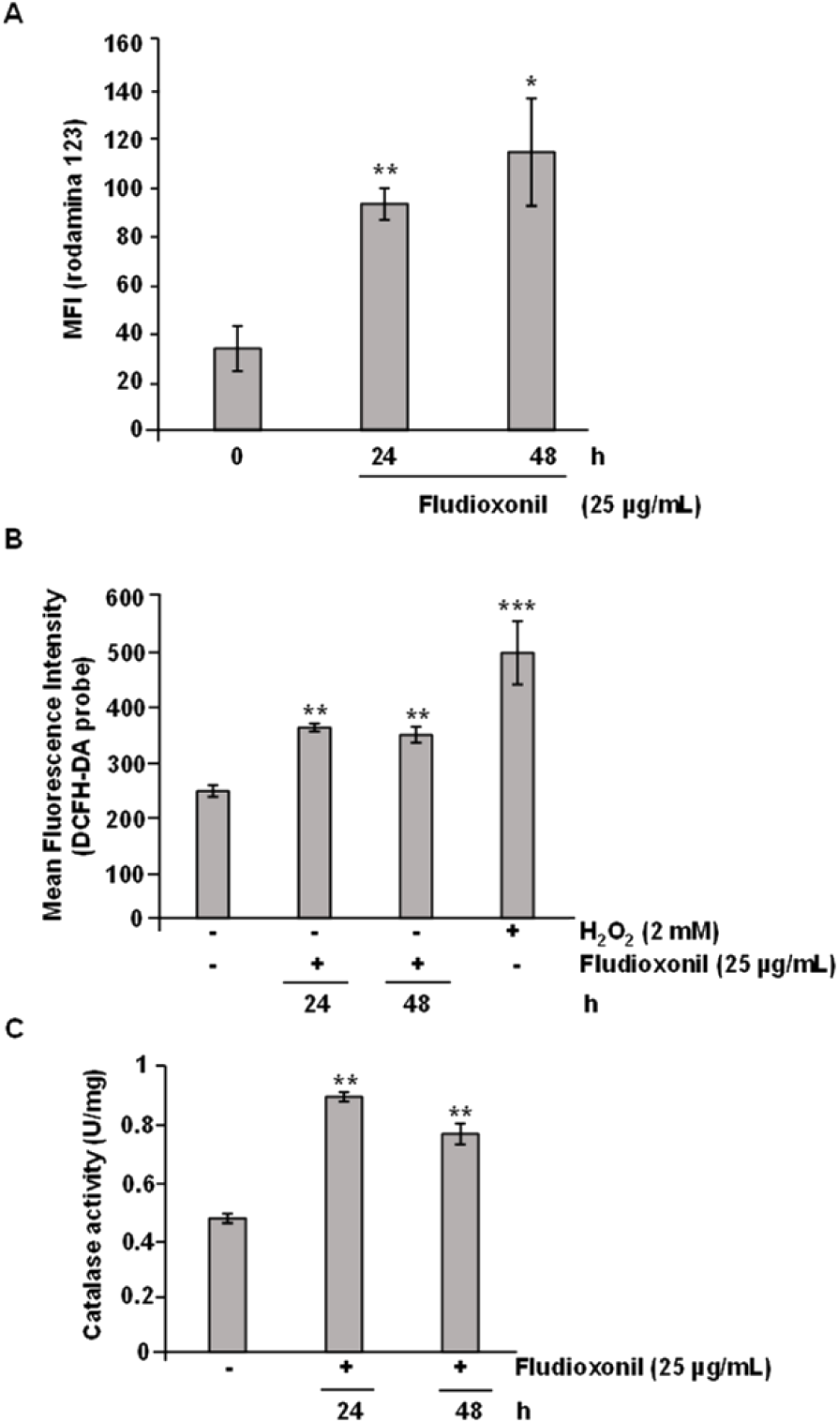
Evaluation of ROS levels and catalase activity of after treatment with fludioxonil. yeasts (1 × 10^6^) were grown in the presence of fludioxonil for 24 and 48 h at 37 °C. (**A**) To detect intracellular ROS, the yeasts were labeled with 10 μM DCFH-DA in the dark for 30 min with gentle shaking (120 rpm), and then the fluorescence reading was performed. H_2_O_2_ (2 mM) was used as a positive control of the reaction. (**B**) The enzymatic activity was measured using the total protein extract (24 and 48 h). The results are expressed as specific activity (catalase units/μg of protein). * *p* ≤ 0.05, ** *p* ≤ 0.01 and *** *p* ≤ 0.001.

Mitochondrial function changes can also impact cellular ROS production.^42^ Therefore, we examined ROS levels in Pb18 yeast cells treated with fludioxonil. As shown in Figure 5B, ROS production was higher in cells treated with fludioxonil compared to the untreated control cells (Figure 5B). Additionally, we measured the activity of catalase in the yeast cells exposed to fludioxonil. This assay revealed approximately a 2-fold increase in enzymatic activity in the fludioxonil-treated samples (Figure 5C). These results suggest that fludioxonil enhances ROS production. Conversely, the increased catalase activity indicates that the fungus adapts to this oxidative stress to restore a balanced redox state.

### Proteomic Analysis of the Cell Wall Proteins-Enriched Fraction of Pb18 Yeast Cells under Fludioxonil Exposure Reveals Remodeling

We investigated the cell wall-enriched proteome of treated with fludioxonil to better understand how this compound affects the composition and remodeling of the fungal cell wall. Given the crucial role of fludioxonil in modulating the cell wall in host interaction and immune evasion,^19^ changes in its protein profile may reveal important antifungal targets and mechanisms of drug action. To achieve this, we performed an enrichment of cell wall proteins using biotin pull-down. The proteomics approach to this kind of preparation is somewhat challenging if biotin is not completely removed from the proteins and might damage the chromatographic columns. The ion chromatograms, ion distribution, and precursor charge state distribution are shown in Figure S1. Using a similar methodology as previously described,^30^ we were able to identify over 1000 proteins per sample in the fraction enriched with cell wall proteins (Figure 6A). As expected because of the methodology used here, the sparsity profile is higher than that of the cell lysates, both per sample and per protein (Figure 6B,C).

**Figure 6:**
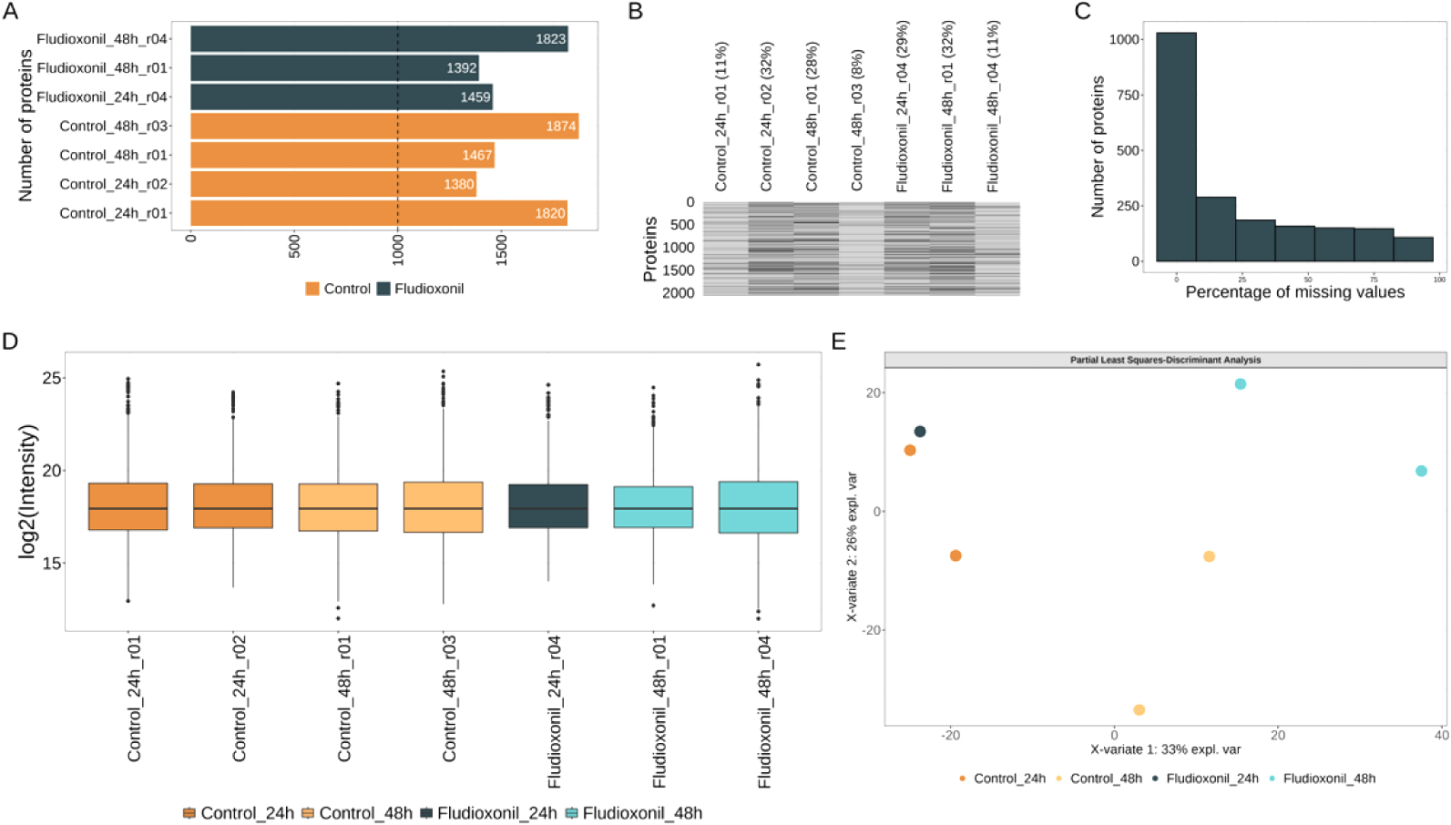
(A) Number of identified proteins per sample. Dashed line marks 1000 proteins. (B) Sparsity map showing the proportion of missing values per sample. (C) Sparsity distribution per protein. (D) Distribution of the normalized intensities for cell wall proteins enriched from Pb18 yeast cells. (E) Sparse partial least-squares-discriminant analysis for the fraction enriched with cell wall proteins.

For this kind of sample preparation, we decided not to reduce the sparsity of the data and keep only the chromatographically consistent samples for protein quantification. After normalization of the protein abundances (Figure 6D) we performed the sparse PLS-DA analysis to discriminate between the groups. We can explain 33% of the variance in the first component and 26% in the second component (Figure 6E). The power to discriminate samples was not as high as the cell lysate samples.

In the fraction enriched with cell wall proteins we detected PCI-containing domain protein (PADG_02525) as significantly decreased 24 h after fludioxonil exposure. This proteasome, COP9, initiation factor 3 composes a signalosome that could suppress the activity of the scaffold protein cullin, avoiding the proteolytic processing of other proteins.^43^ Since the PCI-containing protein domain decreased considerably (log2FC < −5), the cullin suppression mechanism probably did not occur, leaving room for the ubiquitin-proteasome pathway to take place. On the other hand, NADH:flavin oxidoreductase/NADH oxidase N-terminal domain-containing protein (PADG_06196) was significantly increased (log2FC > 3) after 24 h of fludioxonil exposure (Figure 7A,B), once again pointing to an oxidative stress response detected in the fraction enriched with cell wall proteins.

**Figure 7:**
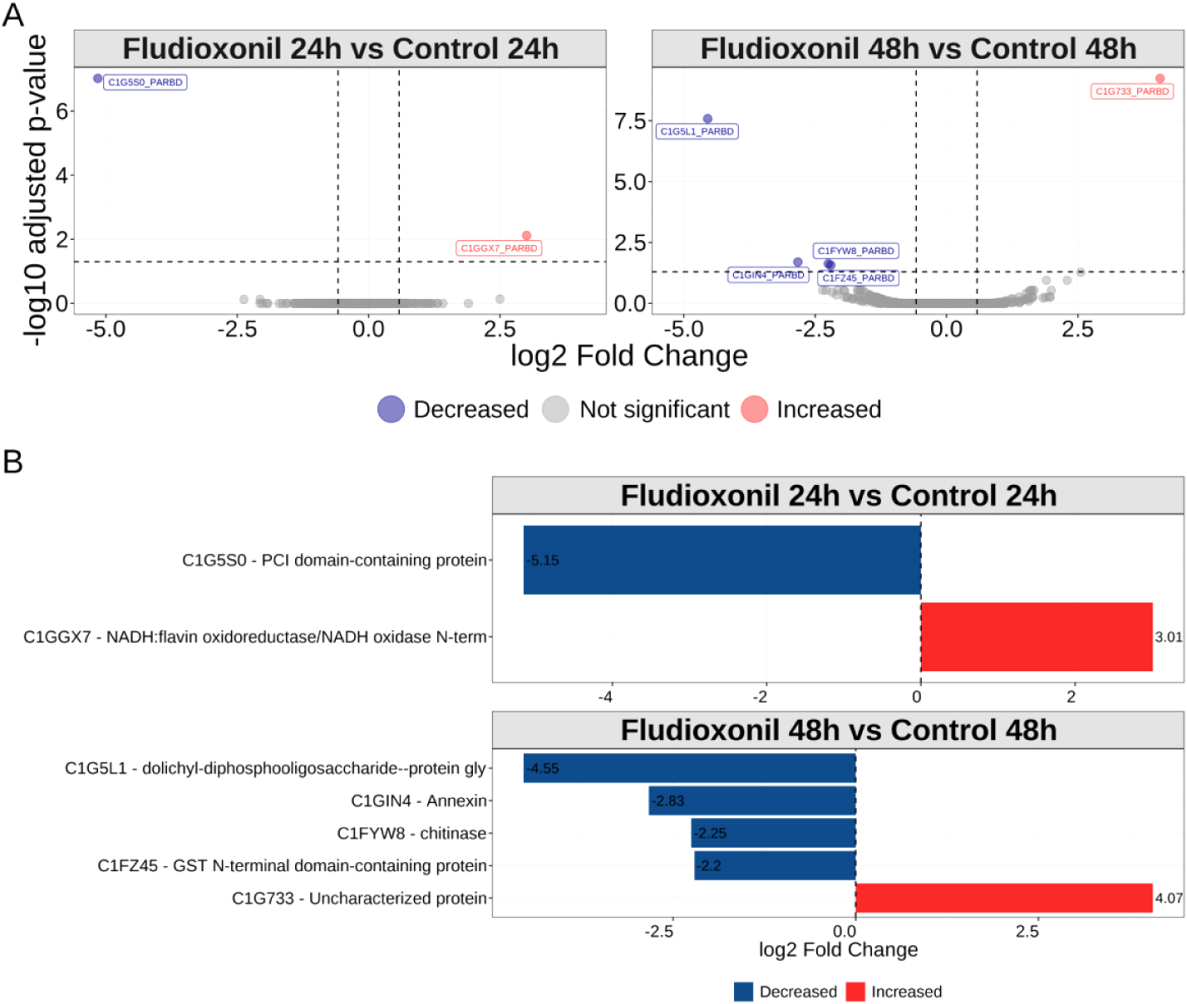
(A) Volcano plot showing the quantification for cell wall proteins enriched fraction under Fludioxonil exposure. Proteins were considered differentially abundant when the absolute log2FC > 0.58 and adjusted p-value δ 0.05. (B) Summary of the differentially abundant proteins with UniProt accession number. Blue is decreased and Red is increased.

Interestingly, 48 h after fludioxonil exposure, a remarkable decrease in the mannosyltransferase (PADG_02472) abundance was observed suggesting that this *O*-glycosylation event on Ser/Thr residues that is determinant for the folding and solubility of proteins was impaired. Chitinase (a glycosidase) was also decreased, suggesting an accumulation of chitin on the cell wall. The decrease in the annexin abundance suggests a perturbation in calcium signaling, while the GST-N-terminal containing protein decrease is suggestive of impaired response to oxidative stress. The only significantly increased protein identified in Pb18 yeast cells 24 h after fludioxonil exposure was the ribonuclease (PADG_02988). This enzyme is reported to be present in yeast exosomes as an important tool for RNA processing and decay.^44^

In the analysis of the cell wall-enriched proteome fraction, all major moonlighting proteins described in the literature, present in the cell wall of and other fungi, were identified. However, only the 14–3–3 protein (PADG_04056) showed a decrease in levels compared to the control samples. The other moonlighting proteins found in the cell wall of Pb18 did not show changes when comparing the fludioxonil-treated and untreated samples (data not shown).

Additionally, we detected key proteins directly involved in the synthesis and remodeling of the cell wall in the cell-wall-enriched protein fraction. In this group, 26 proteins were identified, 11 showing reduced levels in the fludioxonil-treated sample compared to the control (Table 2). This group includes three 1,3-β-glucosidases (PADG_12370, PADG_02862, and PADG_07615), chitinase (PADG_00994), glycosidase (PADG_03691), and α-1,3-glucan synthase (PADG_03169). Only two upregulated proteins were detected, both involved in chitin synthesis (PADG_07911 and PADG_07979) (Table 2). These data suggest that fludioxonil induces alterations in the cell wall composition of .

**Table 2:** List of Proteins Related to Cell Wall Biosynthesis and Remodeling Detected in the Enriched Fraction of Exposed with Fludioxonil

**Gene**	**Protein name**	**log2FC**
PADG_00994	Chitinase	–2,251
PADG_12370	Glucan endo 1,3-beta-glucosidase	–1,657
PADG_07615	Glucan 1,3-beta-glucosidase	–1,300
PADG_03691	Glycosidase	–1,386
PADG_04061	α-1,2-Mannosidase	–1,16
PADG_03169	α-1,3-glucan synthase	–0,9345
PADG_08619	BIG protein	–0,858
PADG_05612	Mannosyltransferase	–0,819
PADG_01648	1,2 α-galactosyltransferase	–0,781
PADG_02862	Glucan 1,3- β-glucosidase	–0,750
PADG_12307	GH18	–0,685
PADG_12130	Chitin synthase	–0,511
PADG_12426	1,4 α-glucan branching	–0,376
PADG_04497	Mannan polymerase II (ANP1)	–0,240
PADG_03430	Mannan polymerase ANP1	–0,156
PADG_08156	CFW hypersensitive	–0,150
PADG_02145	1,4 α-glucan phosphorilase	–0,087
PADG_03306	MMN9	0,307
PADG_07913	Chitin synthase	0,3373
PADG_06438	Chitin synthase	0,440
PADG_05937	Chitin synthase activator	0,490
PADG_07911	Chitin synthase	0,953
PADG_07979	Chitin biosynthesis protein (CHS5)	0,8317

## Discussion

Using a label-free proteomic approach we identified various proteins that were up- and down-regulated in yeast cells in response to fludioxonil treatment. Mass spectrometry analysis resulted in the identification of 3937 (average number of identifications in the control condition) and 4211 (average number of identifications in the treatment condition) proteins of . The present study offers evidence at the protein level for the existence of over 4000 sequences in the database. This number of identified proteins is an excellent result, considering that the genome (Pb18 isolate) contains 8583 putative genes (https://fungidb.org/), hence our data represent approximately 50% of the total of encoded proteins. Compared to other proteomic studies of this fungus, the identification coverage has ranged between 13.9 and 34% relative to the fungal genome.^45,46^ Most proteomic studies typically identify fewer than a few hundred proteins, so our findings represent a significant contribution to the field. This study benefited from the application of DIA, a method that has not been employed in fungal biology studies.

In addition, our panel of differentially abundant proteins identified after 24 h of fludioxonil exposure is consistent with a response to osmotic and oxidative stress. Fludioxonil, a phenylpyrrole fungicide, exerts its antifungal effects by targeting group III hybrid histidine kinases (HHKs), such as Drk1, which are absent in humans but conserved among pathogenic fungi.^14,18,26^ This specificity makes HHKs attractive targets for antifungal therapy. Fludioxonil induces a conformational change in Drk1, converting it from a kinase to a phosphatase, which leads to the dephosphorylation of its downstream target, Ypd1. This effect triggered constitutive activation of the HOG pathway, culminating in fungal cell death.^20^ Moreover, fludioxonil disrupts glycolytic processes by inhibiting triosephosphate isomerase, causing an accumulation of methylglyoxal, which further activates Drk1 and the HOG pathway.^47^ Additionally, the resistance to group III HHK inhibitors, such as fludioxonil, has been linked to high catalase activity,^48^ implying that fungal resistance depends on the ability to scavenge reactive oxygen species. In agreement, we observed an increased abundance of flavin oxidoreductase after 24 h of fludioxonil exposure in the cell wall-enriched fraction. After 48 h, we observed a significant decrease in the GST-N-terminal containing protein. Altogether, these findings suggest that fludioxonil impairs fungal growth and also interferes with virulence-associated pathways, including morphogenesis^14^ and stress responses.^20^ Thus, our results reinforce HHKs as a potential target for the development of innovative and new antifungal compounds.

It is well established that mitochondria are related to oxidative stress in eukaryotic cells. Considering that our proteomic data revealed the presence of proteins involved in oxidative response, we investigated the influence of fludioxonil treatment on mitochondria. A qualitative analysis of mitochondrial membrane potential was conducted in treated with fludioxonil over different periods. The hyperpolarization of the mitochondrial membrane observed in the assay may be related to the inhibition of Drk1 by fludioxonil. Studies have reported changes in plasma membrane potential due to H^+^ influx, along with altered mitochondrial membrane potential in after treatment with another phenylpyrrole derivative.^49^ Similarly, in , fludioxonil-induced hyperpolarization occurred through H^+^ efflux and K^+^ influx, leading to increased membrane potential.^50^ In , studies on mitochondrial metabolism and functional alterations in this organelle are still in their early stages.^51^ A more detailed and in-depth analysis of mitochondrial function and activity will be further explored in our model to understand the biochemical mechanisms involved in oxidative stress and fludioxonil.

Previous work from our group has demonstrated that fludioxonil modulates cell wall composition.^19^ The identification of proteins related to the yeast cell wall in total proteomic analysis after fludioxonil treatment confirms our previous functional studies. The evaluations of proteins differentially abundant in the yeast cell wall identified that enzymes related to carbohydrate metabolism were among the most affected. The decrease in both mannosyltransferase and Chitinase could be related since most proteins are known to be glycosylated to maintain solubility and activity in eukaryotic cells. *O*-mannosylation is a post-translational modification that mainly affects proteins localized in the yeast cell wall.^52^ The oxidative/osmotic stress response induced by fludioxonil exposure may be affecting the cell wall carbohydrate composition by decreasing *O*-mannosylated proteins and accumulating chitin.

In addition, the Hog1MAP kinase pathway plays a central role in modulating fungal cell wall composition. In , deletion of the HOG1 gene increases β-glucan exposure and decreases chitin levels, supporting the regulatory role of Hog1 in maintaining cell wall architecture.^53^ Hog1 also influences the expression of genes related to cell wall organization, affecting the fungus’s ability to adapt to environmental stresses and maintain cell wall integrity.^54^ These findings underscore the role of fludioxonil in modulating the Hog1MAPK pathway, which may lead to changes to downstream alterations in the composition and organization of fungal cell wall components.

The proteomic analysis of enrichment cell wall proteins demonstrated that surface proteins affected by fludioxonil are involved in cell wall biosynthesis and remodeling. It is known that exhibits differences in cell wall components among its various morphologies.^55^ The mycelial form is rich in β-1,3- and β-1,6-glucan, while the yeast form contains higher levels of α-1,3-glucan and chitin.^55^ This differential composition is crucial for fungal survival within the host, as the α-1,3-glucan is linked to virulence.^56^ In addition, masking of immunogenic β-1,3-glucan by α-1,3-glucan facilitates immune evasion through recognition by the dectin-1 receptor.^25^ The morphological switch between the fungal forms is believed to be an additional evasion strategy against phagocytic cells and the recognition mechanisms of cell wall components,^25^ highlighting the critical role of cell wall architecture in immune recognition.

We observed reduced levels of three 1,3-β-glucosidases (enzymes that hydrolyze β-glucan polymers) and a Chitinase, as well as an increased abundance of two chitin synthases. These changes suggest that fludioxonil promotes cell wall remodeling, enhancing the exposure of key pathogen-associated molecular patterns (PAMPs) and possibly facilitating host immune recognition. In this context, it is important to consider the role of cell surface proteins, which cooperate with polysaccharide components to maintain cell wall integrity and influence the outcome of host–pathogen interactions.

Proteins, in combination with other cell wall components on the surface of fungi, play a crucial role in protecting the fungus against environmental stress and host defense responses during infection.^57–61^ In and other fungi, several surface-associated proteins function as moonlighting proteins, contributing not only to cellular metabolism but also to host–pathogen interactions. These proteins, including GAPDH, Gpi1, Tpi, Gpd2, enolase, Adh, 14–3–3, malate synthase, and heat shock proteins (Hsp60, Hsp70),^62,63^ represent important virulence factors. While many of these were identified in our surface proteome analysis, only the 14–3–3 protein showed significantly decreased abundance after fludioxonil treatment. This protein acts as an adhesin in ,^64^ and studies using murine and models demonstrated that reduced expression of 14–3–3 correlates with decreased virulence.^65,66^ Therefore, its modulation by fludioxonil is a particularly intriguing finding that deserves further investigation.

Although our findings provide relevant insights into the effects of fludioxonil on , we acknowledge that additional experimental conditions could have strengthened the conclusions. In particular, the inclusion of multiple time points and a broader range of fludioxonil concentrations would have allowed a more comprehensive understanding of the temporal dynamics and dose-dependent effects of this compound. Nonetheless, the results presented here open important avenues for future research, especially regarding the modulation of virulence-associated pathways and the potential of fludioxonil as an antifungal strategy.

In summary, our study demonstrates that fludioxonil modulates intracellular pathways and significantly alters the cell wall composition of . The regulation of proteins involved in cell wall remodeling and stress responses indicates broad interference with fungal physiology. Reduced levels of 14–3–3 protein, changes in mitochondrial membrane potential, and increased exposure to β-glucan and chitin support the hypothesis that fludioxonil interferes with virulence mechanisms. These findings may provide support for future studies that aim to exploit HHKs and cell wall biosynthetic pathways as potential targets for antifungal therapy.

## Supplementary Material



## Data Availability

All raw data files have been made publicly available via the MassIVE repository under the identifier MSV000096800 (ftp://MSV000096800@massive.ucsd.edu).

## Notes

Figure S1. chromatographic and mass spectrometry data from the enriched cell wall protein fraction of Pb18 yeast cells, including ion chromatograms, retention time distribution, and precursor charge states (PDF)
