# Supplementary material for: Proteomic Changes in Induced by Fludioxonil
Source: J Proteome Res. 2025 Jun 13;24(7):3310–23. doi: 10.1021/acs.jproteome.5c00038 (PMC12235711; doi:10.1021/acs.jproteome.5c00038)
Supplement: Supplementary file 1 [file pr5c00038_si_001.pdf]

SUPPORTING INFORMATION

Proteomic changes in *Paracoccidioides brasiliensis* induced by fludioxonil

Pedro Henrique Corteletti Manfio[1]; Wilson Dias Segura[2]; Marina Valente Navarro[2]; Rogéria Cristina Zauli[1]; Patricia Santos Lopes[1]; Solange M. T. Serrano[3]; Alison Felipe Alencar Chaves[3*]; Wagner Luiz Batista[1,2*]

[1] Department of Pharmaceutical Sciences, Federal University of São Paulo, 09913-030 Diadema, SP, Brazil.

[2] Department of Microbiology, Immunology and Parasitology, Federal University of São Paulo, 04023-062 São Paulo, SP, Brazil.

[3] Laboratory of Applied Toxinology, Center of Toxins, Immune-response and Cell Signaling, Butantan Institute, 05503-900 São Paulo, SP, Brazil.

Summary

Figure S1 – Chromatographic and mass spectrometry data from the enriched cell wall protein fraction of Pb18 yeast cells..Page S1

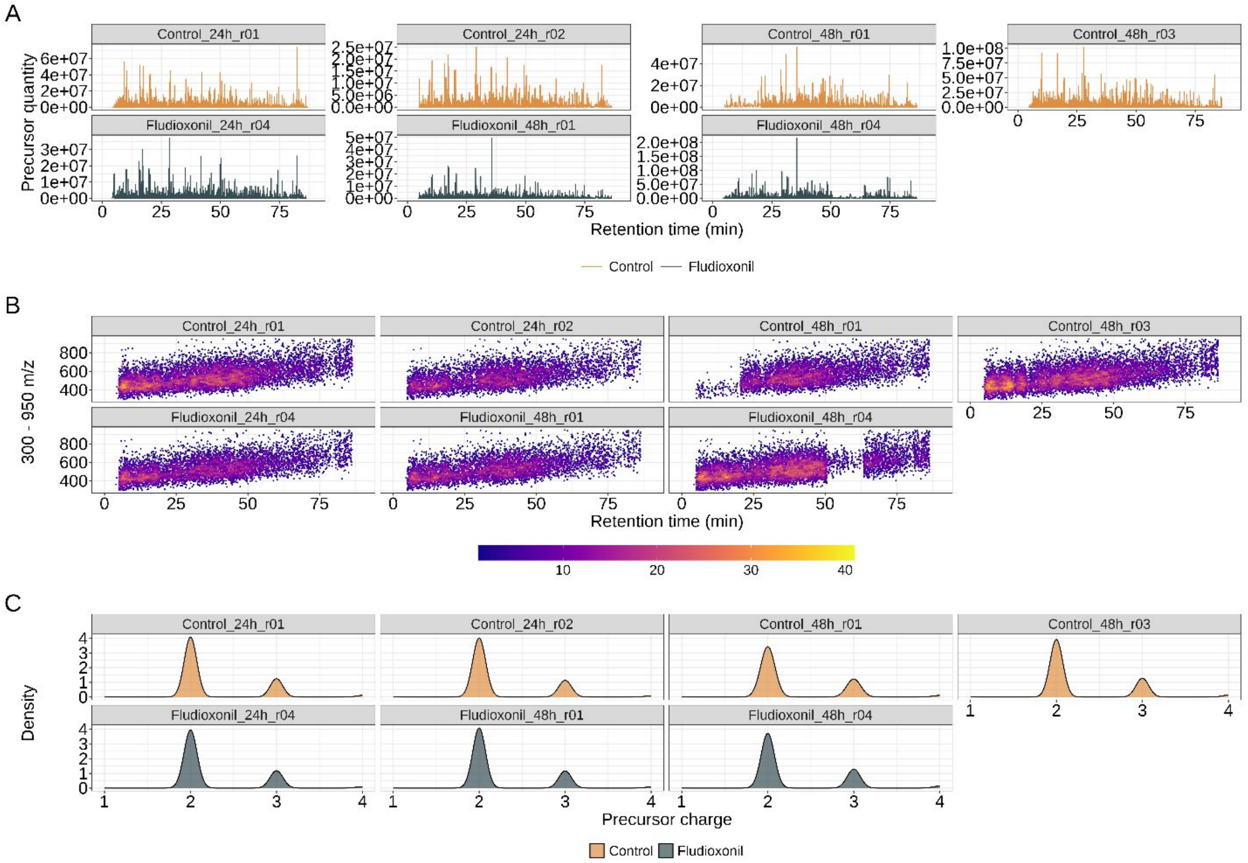

Figure S1. (A) Ion chromatograms from cell wall proteins enriched fraction from Pb18 yeast cells. **(B)** Ion distribution over the retention time for each LC-MS/MS run. **(C)** Charge state distribution for precursors.
